# Strategies for Forecasting Long COVID in the Active Component U.S. Military

**Published:** 2025-10-01

**Authors:** Mark L. Bova, Tara N. Palmore, Guoqing Diao, Jamaal A. Russell, Manya Magnus

**Affiliations:** Integrated Biosurveillance Branch, Armed Forces Health Surveillance Division, Silver Spring, MD: Dr. Bova, Dr. Russell; Department of Epidemiology, Milken Institute School of Public Health, George Washington University, Washington, DC: Dr. Bova, Dr. Magnus; Department of Medicine, School of Medicine and Health Sciences, George Washington University: Dr. Palmore; Department of Biostatistics and Bioinformatics, Milken Institute School of Public Health, George Washington University: Dr. Diao

## Abstract

Long COVID, or post-acute coronavirus disease syndrome, represents a potentially serious threat to military readiness. Forecasts of future long COVID diagnoses could help prepare senior leaders for disruptions. Few studies predicting the incidence of long COVID have been published to date, however. Using existing COVID-19 and long COVID diagnoses, as well as demographic and outpatient encounter data, 1- to 6-month ahead and full 6-month forecasts were generated using time series and machine learning models trained on various covariate data. Forecasting models generated accurate predictions of long COVID diagnoses up to 6 months ahead of the forecasted date. Several model and covariate combinations were within 5% of the observed number of diagnoses over the full 6-month testing period, while monthly forecasts of long COVID diagnoses had median absolute percentage errors ranging from 3% to 10% for the best performing model combinations. Simple forecasting models and distribution-based forecasts that utilize existing clinical databases can provide accurate predictions of incident long COVID up to 6 months in advance and can be used to prepare for the burden of new long COVID diagnoses.

Long COVID, or post-acute coronavirus disease syndrome, has been well studied in the general population, although it has not been well established in the U.S. military. Internal, not yet published Defense Medical Surveillance System (DMSS) data from active component U.S. service members diagnosed with coronavirus disease 2019 (COVID-19) from January 2020 through December 2022 indicate that symptoms of long COVID may be present in up to 20% of service members, with cardiac symptoms in approximately 8% and respiratory symptoms in approximately 5% of service members (unpublished). Another study of active duty service members with COVID-19 diagnoses from March 2020 to November 2021 found cardiac symptoms in nearly 2% of service members more than 30 days after COVID-19 diagnosis. ^1^ At best, mild symptoms of long COVID could disrupt force readiness by causing unplanned training limitations and absences, while more severe symptoms could result in long-term disability or even death. It is, therefore, critical for senior U.S. Department of Defense (DOD) leaders to anticipate the burden of long COVID in advance to prepare for potential disruptions and to anticipate impacts on the military health care system resources.

Infectious disease forecasting, especially for influenza, has been conducted for decades. Various mechanistic, statistical, and time series models have been used for forecasting, as well as combined ensemble models. The U.S. Centers for Disease Control and Prevention (CDC) hosts annual forecasting challenges for influenza and COVID-19 aimed at predicting short-term incidence of cases and hospitalizations. ^2^ The CDC has found that ensemble models tend to be more stable and accurate for multiple forecasting locations and targets than individual models, including COVID-19 forecasting. ^3 - 4^

**What are the new findings?**

Accurate predictions of long COVID cases over a 6-month period were achieved by utilizing existing COVID-19 case and out-patient encounter data from January 1, 2020, through December 31, 2022.

**What is the impact on readiness and force health protection?**

Long COVID symptoms can cause disruptions to military readiness and prevent a healthy force, especially after surges in COVID-19 cases. The ability to use existing data sources to accurately predict future cases of long COVID allows senior leaders to anticipate and prepare for potential changes in the availability of service members.

Long COVID is a long-term, post-infectious process of COVID-19, however, that is not contagious and requires a person to both be infected with COVID-19 and develop symptoms of long COVID after a specified period. Traditional time series methods for forecasting short-term COVID-19 and other respiratory disease activity may not be useful for forecasting long COVID cases, and little research has been published to date on efforts to predict the incident number of long COVID diagnoses utilizing existing case data, especially within the military population. Studies using clinical data in civilian populations found various models to be reasonably accurate, with AUROC (area under a receiver operating characteristic) values between 0.74 and 0.895. ^5 - 7^ Attempts have been made to use time series models to forecast incident cases of other diseases with long follow-up periods, such as Lyme disease, using clinical data, with mean absolute percentage errors around 8%. ^8^

The purpose of this study was to develop predictive models to forecast future long COVID diagnoses and to compare the predictions of each model against observed long COVID diagnoses. To achieve this aim, this study utilized a cohort of COVID-19 cases, linked demographic and medical records, and longitudinal health encounter data.

## Methods

The protocol for this study was approved by both the George Washington University Committee on Human Research Institutional Review Board and the Component Office for Human Research Protections of the Defense Health Agency Office of Research Protections.

### Study population

The study population included a cohort of 464,356 active component U.S. service members with a confirmed case of COVID-19 at a U.S. military hospital or clinic, from January 1, 2020 through December 31, 2022. The U.S. active component includes full-time, active duty service members but excludes reservists or National Guard members.

Data were obtained from a master list of COVID-19 cases, defined as having either a positive SARS-CoV-2 (severe acute respiratory syndrome coronavirus 2) nucleic acid or antigen test or a COVID-19 reportable medical event (RME) in the Disease Reporting System Internet (DRSi) maintained by the Armed Forces Health Surveillance Division (AFHSD). The master list includes information relevant to a service member's COVID-19 event, including vaccinations, re-infection status, and hospitalization.

### Exposures and covariates

Covariates of interest in this study focused on measures of COVID-19 activity, including COVID-specific, COVID-like illness (CLI), and post-acute sequelae of COVID-19 (PASC) outpatient encounters, as well as risk factors for long COVID. Risk factors included sex, age, race and ethnicity, rank, COVID-19 hospitalization status, COVID-19 re-infection status, and COVID-19 vaccination status.

Demographic information for each COVID-19 case in the master positive list was taken from the Defense Medical Surveillance System (DMSS), a DOD-maintained database of health information that includes personnel, medical, immunization, pharmacy, health assessment, laboratory, and deployment data. ^9^ Monthly aggregated outpatient encounters by military hospital or clinic were downloaded from the DOD Electronic Surveillance System for the Early Notification of Community-based Epidemics (ESSENCE).

COVID-specific encounters were defined as any outpatient encounter with a discharge diagnosis containing the ICD-10 codes U07.1 or J12.81, while PASC encounters were defined as those containing the ICD-10 code U09.9. The CLI encounter definition is provided in **Supplementary Table 1** .

**SUPPLEMENTARY TABLE 1.** PASC Diagnostic Categories and ICD-10 Diagnosis Codes

Mental Health	
Anxiety	F4323, F419, F411, F418, F4322
Insomnia	G4700, F51
PTSD	F4312
Major depression	F321, F329, F331, F332, F339, R45851
Neurological	
Headache	R51
Taste loss	R430, R431, R432, R438, R439
Seizure	R5600, R5601, R569, G40001, G40009, G40011, G40019, G40101, G40109, G40111, G40119, G40201, G40209, G40211, G40219, G40301, G40309, G40311, G40319, G40401, G40409, G40411, G40419, G40501, G40509, G40801, G40802, G40803, G40804, G40811, G40812, G40813, G40814, G40821, G40822, G40823, G40824, G4089, G40901, G40909, G40911, G40919, G40A01, G40A09, G40A11, G40A19, G40B01, G40B09, G40B11, G40B19
Blurred vision	H53
Fatigue	R53
Memory loss	R413
Cognitive dysfunction	R410, R411, R412
Vertigo	H8110, H8111, H8112, H8113, H8141, H8142, H8143, H8149
Respiratory	
Cough	R05
Short of breath	R0602, R0600, R0609
Pulmonary embolism	I2699
Asthma	J45909, J4520, J45901, J4530, R062
Cardiac	
Chest pain	R079, R0789, R072
Palpitations	R002
Atrial fibrillation	I4891, I480
Syncope	R55
Tachycardia	R000
Heart failure	I509, I517
Bradycardia	R001
Myocardial infarction	I21, I22
Cerebral infarction, stroke	I63
Post-exertional malaise	T733
Myocarditis	I40, I41, I514, B3322
Pericarditis	I30, I32, B3323

Abbreviations: PASC, Post Acute Sequelae of COVID-19; ICD-10, International Classsification of Diseases, 10th Revision; PTSD, post-traumatic stress disorder.

### Case definition

The outcome of interest, long COVID, was assessed using the PASC definition developed and validated by the Defense Centers for Public Health–Portsmouth (DCPHP). Briefly, the definition requires a service member to have a positive SARS-CoV-2 nucleic acid test or a confirmed COVID-19 RME, and an International Classification of Diseases, 10th Revision (ICD-10) code from 1 of the mental health, neurological, cardiac, or respiratory diagnostic groups from 4 to 52 weeks after the COVID-19 event. Diagnostic groups and their ICD-10 codes are shown in **Supplementary Table 2** . A service member must not have the same diagnosis within that specific diagnosis group within 1 year prior to the COVID-19 event. Inpatient and outpatient datasets from DMSS were used to identify incidence of long COVID in this population.

**SUPPLEMENTARY TABLE 2.** ESSENCE COVID-like Illness (CLI) Definition

Diagnosis Description	ICD-10 Code
Coronavirus, unspecified	B34.2
SARS-associated coronavirus as cause of disease classified elsewhere	B97.21
Other coronavirus as cause of disease classified elsewhere	B97.29
Acute nasopharyngitis, common cold	J00
Acute upper respiratory infection, unspecified	J06.9
Pneumonia due to SARS-associated coronavirus	J12.81
Other viral pneumonia	J12.89
Viral pneumonia unspecified	J16.8
Pneumonia in diseases classified elsewhere	J17
Bronchopneumonia, unspecified organism	J18.0
Lobar pneumonia, unspecified organism	J18.1
Other pneumonia, unspecified organism	J18.8
Pneumonia, unspecified organism	J18.9
Acute bronchitis due to other specified organisms	J20.8
Acute bronchitis, unspecified	J20.9
Unspecified acute lower respiratory infection	J22
Bronchitis, not specified as acute or chronic	J40
Acute respiratory distress syndrome	J80
Idiopathic interstitial pneumonia not otherwise specified	J84.111
Cough	R05
Dyspnea	R06.0
Dyspnea, unspecified	R06.00
Shortness of breath	R06.02
Acute respiratory distress	R06.03
Other forms of dyspnea	R06.09
Anosmia	R43.0
Ageusia	R43.2
Fever, unspecified	R50.9
2019-nCoV acute respiratory disease, COVID-19, virus identified	U07.1

Abbreviations: ESSENCE, Electronic Surveillance System for the Early Notification of Community-based Epidemics; SARS, severe acute respiratory syndrome; 2019-nCov, 2019 novel coronavirus; COVID-19, coronavirus disease 2019.

### Analyses

This study focused on longitudinal forecasts of long COVID in the U.S. active component population. To facilitate time series forecasting, long COVID, COVID-19, and outpatient encounter data sets were converted into time series by aggregating the monthly numbers of cases and encounters. COVID-19 cases were additionally stratified by risk factor. The number of monthly cases and encounters were plotted together to visualize the relationship between each metric and the outcome of long COVID.

The data were divided into training and testing datasets. The training data-set included data from January 1, 2020 through June 30, 2022, and the testing data-set included data from July 1, 2022 through December 31, 2022. Using the training data, 3 models were fit with long COVID diagnoses as the outcome: autoregressive integrated moving average (ARIMA), neural network, and vector autoregressive (VAR), in addition to an ensemble model that represented the average of the other 3 models. Different versions of each model were fit, with 21 in total that featured different data lags (unlagged, 3-month lag, and 6-month lag) and covariate data including PASC encounters, COVID-19 cases, COVID-specific encounters, CLI encounters, and demographics (age, sex, race and ethnicity, rank, re-infection status, hospitalization status, and vaccination status). All model and covariate combinations are shown in **Table 1** . Model fit statistics were assessed for the training period, including Akaike information criterion (AIC), sigma ^2^ (variance of forecast errors), root mean squared error (RMSE), and median absolute percent error (MAPE).

**TABLE 1.** Median Ensemble Model Fit Statistics, by Training Covariates and Lagging

Combination	AIC ^a^	Sigma ^2 b^	RMSE	MAPE
Base (no covariates)	331.5	250,962.5	491.2	10.1
PASC encounters				
	427.9	267,422.6	525.4	18.0
COVID-19 cases				
No lag	327.7	124,189.8	313.6	8.8
3-month lag	421.9	250,262.5	645.6	19.6
6-month lag	331.5	271,967.1	528.5	11.5
COVID-19 encounters				
No lag	331.4	211,062.8	445.8	11.0
3-month lag	422.7	277,948.1	643.0	20.1
6-month lag	331.6	202,544.6	441.7	9.8
CLI encounters				
No lag	331.6	217,329.0	455.5	10.5
3-month lag	421.4	258,492.6	630.0	17.9
6-month lag	420.5	254,913.9	621.4	17.3
COVID-19 cases, COVID-19 encounters and CLI encounters				
No lag	327.9	156,411.2	392.1	8.3
3-month lag	425.3	284,716.0	610.4	16.3
6-month lag	326.4	201,484.5	431.9	9.9
Demographics: age, sex, race and ethnicity, rank, re-infection status, hospitalization status				
	463.8	223,839.5	210.1	14.9
COVID-19 cases, COVID-19 encounters, CLI encounters and demographics				
No lag	470.2	350,118.4	262.6	75.8
3-month lag	494.5	856,769.3	391.9	93.3
6-month lag	485.7	623,760.2	334.1	112.3
All: PASC encounters, COVID-19 cases, COVID-19 encounters, CLI encounters and demographics				
No lag	465.1	299,529.7	231.8	56.6
3-month lag	-103.8	0.0	0.4	0.05
6-month lag	422.1	64,483.4	108.0	85.7

Abbreviations: AIC, Akaike information criterion; RMSE, root mean squared error; MAPE, median absolute percent error; PASC, post-acute sequelae of COVID-19; COVID-19, coronavirus disease 2019; CLI, COVID-like illness.

a Not available for neural network (NNET) model.

b Not available for vector autoregressive (VAR) model.

Models showing the best fit with the training data were selected for forecasting, including the models with all COVID-19 metrics and those with all metrics. The models using PASC encounters were also included for forecasting. Several baseline models were also created for comparison.

First, a seasonal NAÏVE was calculated using a 5-month lag of COVID-19 cases and 22% of COVID-19 cases diagnosed with long COVID in the cohort. The lag parameter represented the average time in months from the COVID-19 event date to the long COVID diagnosis date in the cohort, and the long COVID incidence parameter represented the percentage of COVID-19 cases diagnosed with long COVID in the sample.

Second, the distribution of the time from the COVID-19 event date to the long COVID diagnosis date in the cohort was estimated to be a Weibull distribution with a shape parameter of 1.56 and scale parameter of 5.81. A distribution of diagnosis times was calculated using the Weibull parameters, the long COVID incidence parameter described, and a minimum diagnosis time of 1 month and maximum of 12 months. The calculated distribution was applied to the time series of COVID-19 cases to create an estimate of expected long COVID diagnoses by month.

Similarly, an adjusted Weibull prediction was created using a long COVID incidence parameter that varied by risk factor. Based on factor-specific incidence of long COVID in the cohort, the parameter was estimated for sex (32% for females, 20% for males), race and ethnicity (21% for Asian, 22% for Hispanic, 27% for non-Hispanic Black, 21% for non-Hispanic White, 22% for ‘other’), age group (19% for <20, 21% for 20-34, 27% for 35-39, 30% for 40-44, 31% for 45+), rank (23% for enlisted, 19% for officers), COVID-19 reinfection status (22% for first infection, 26% for re-infection), and COVID-19 hospitalization status (22% for not hospitalized, 43% for hospitalized). The average calculated distribution was applied to the time series of COVID-19 cases to create an estimate of expected long COVID diagnoses by month.

Lastly, an ensemble model was calculated as the average of all models for each covariate and lag combination as well as overall.

Two sets of forecasts were generated for each model combination. First, the number of long COVID diagnoses during the entire 6-month testing period was forecasted using the training dataset. Second, for each month during the testing period (July–December), forecasts were generated for each remaining month in the testing period (through December 2022). Models used data through the end of the previous month for training. For example, data through July 31, 2022, were used to generate forecasts for August, September, October, November, and December 2022. Data through August 31, 2022 were used to generate forecasts for September, October, November, and December 2022. This continued through the end of the testing period. Seasonal naïve and ensemble forecasts were generated in both quantile and point formats to facilitate evaluation of the complete distribution of the forecasts. Forecasts using the Weibull distribution were only generated as a point forecast.

Forecasts were scored by comparing the predicted number of long COVID diagnoses in a period to the observed number. Monthly point forecasts were scored using a MAPE, and quantile forecasts were scored using a weighted interval score (WIS). Full 6-month point forecasts were scored using percentage error. WIS has been used previously by the CDC for scoring COVID-19 forecasting hub entries. ^10^ All statistical analyses were conducted using R (version 4.1, R Foundation for Statistical Computing, Vienna, Austria), and an alpha (α) level of 0.05 was considered statistically significant.

## Results

**FIGURE 1.**
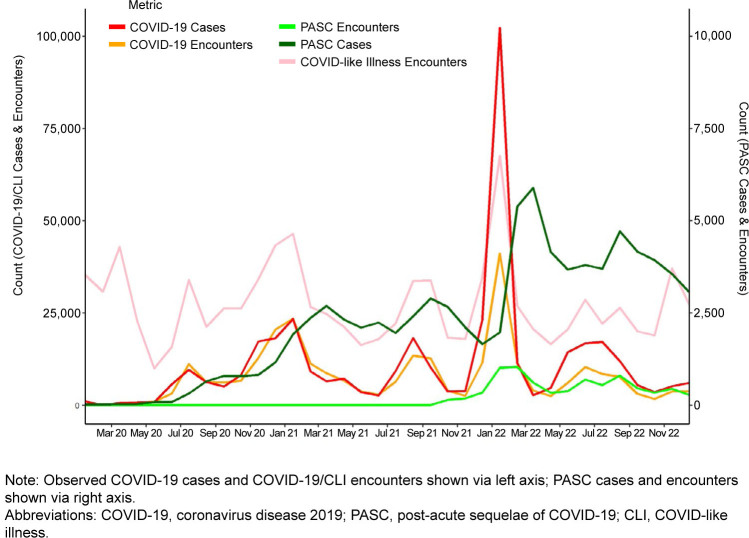
Observed Monthly Numbers of Cases and Encounters, by Metric, 2020–2022

**Table 2** shows demographic characteristics of COVID-19 cases in the training and testing datasets. Datasets were similar by age, race and ethnicity, rank, and COVID-19 hospitalization, although a larger proportion of the testing dataset was female (24.3% vs. 20.4%). COVID-19 re-infections were much more prominent in the testing dataset (19.2% vs. 5.5%), although this was expected, as the testing data were generated nearly 2 years into the COVID-19 pandemic. **Figure 1** shows the time series of observed data used for training and prediction in this study. As expected, incidence of COVID-19 was higher than PASC, with COVID-19 cases peaking between 10,000 and 20,000 monthly cases each summer, and between 25,000 and 100,000 monthly cases each winter, while PASC cases peaked between 2,500 and 6,000 monthly cases. PASC peaks tended to follow peaks in COVID-19 activity by 2 to 3 months.

**TABLE 2.** Demographic Characteristics of COVID-19 Cases During Training and Testing Periods

Variable	Cases			
	Training		Testing	
	No.	%	No.	%
Total	402,352		62,004	
Age, *y*				
<20	27,950	6.9	3,276	5.3
20–24	138,332	34.4	19,323	31.2
25–29	96,233	23.9	15,117	24.4
30–34	62,144	15.4	10,366	16.7
35–39	45,198	11.2	7,766	12.5
40–44	21,242	5.3	3,870	6.2
45+	11,251	2.8	2,286	3.7
Sex				
Female	82,064	20.4	15,050	24.3
Male	320,288	79.6	46,954	75.7
Race and ethnicity				
White, non-Hispanic	203,674	50.6	30,678	49.5
Black, non-Hispanic	70,954	17.6	10,547	17.0
Hispanic	80,899	20.1	12,305	19.8
Asian	15,462	3.8	2,772	4.5
Other	25,311	6.3	4,845	7.8
Unknown	6,052	1.5	857	1.4
Rank				
Enlisted	344,510	85.6	53,362	86.1
Officer	57,842	14.4	8,642	13.9
Re-infection of COVID-19				
First infection	380,256	94.5	50,121	80.8
Re-infection	22,096	5.5	11,883	19.2
Hospitalization for COVID-19				
Not hospitalized	399,367	99.3	61,611	99.4
Hospitalized	2,985	0.7	393	0.6

Abbreviations: COVID-19, coronavirus disease 2019; No., number; *y* , year.

**SUPPLEMENTARY FIGURE 1.**
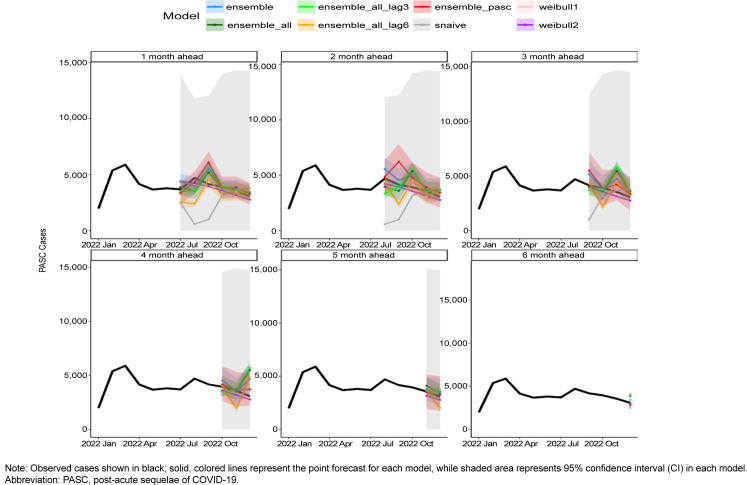
Observed Versus Median Fitted Prediction, by Training Covariates and Data Lag

**Table 1** shows model fit statistics for each combination of trained models during the training period. The lowest AIC was seen for the 3-month lag model containing all covariates (-103.8). This model combination also had the lowest sigma ^2^ (0.0), RMSE (0.4), and MAPE (0.05%) compared to other combinations. Other model combinations with a MAPE below 10% were the unlagged COVID-19 case model (8.8%), 6-month lagged COVID-19 encounter model (9.8%), unlagged all COVID-19 metric model (8.3%), and the 6-month lag all-COVID-19 metric model (9.9%). Graphs of the median fitted predicted values for each model combination and lag compared to observed data are shown in **Supplementary Figure 1** . All models appeared to fit the observed data visually, although the models with all covariates and those with only demographic covariates appeared to fit the data best.

**FIGURE 2.**
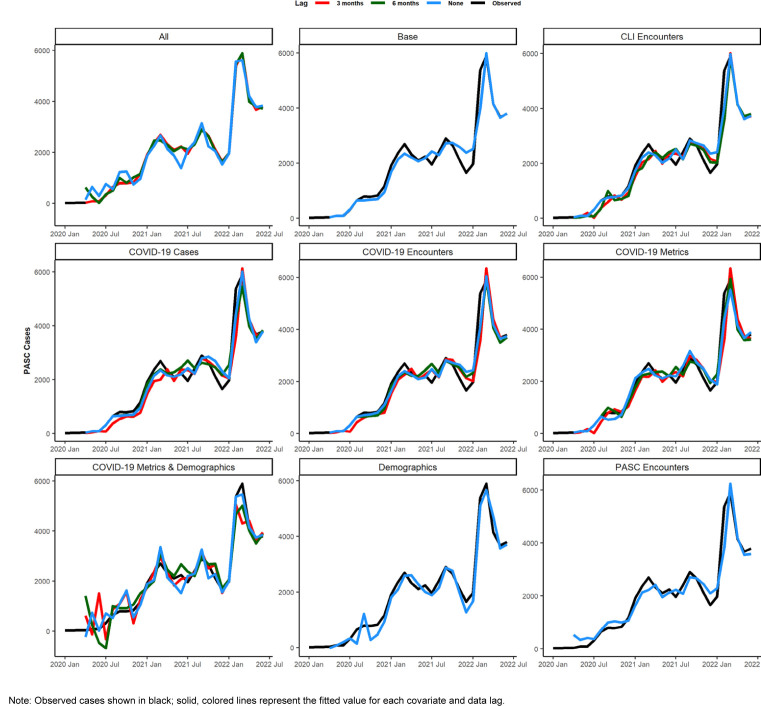
Observed Versus Predicted Value, by Selected Ensemble and Baseline Models and Forecasting Horizon

**Table 3** shows model scoring metrics for each ensemble and baseline model and forecasting horizons. For all forecasting horizons, the ensemble model using PASC encounters had the lowest median MAPE (9.2%) and weighted interval score (WIS) (206.6), followed by the 3-month lag ensemble model using all covariates (11.3% MAPE, 291.0 WIS), and the unadjusted Weibull model (MAPE 11.5%). Model performance varied between the 1-month ahead and 6-month ahead horizons. **Figure 2** shows the observed compared to predicted values for each model and horizon. Ensemble models tended to predict a later peak than what was observed for the 1-month ahead through 3-month ahead forecasts, although this was less severe for the ensemble model using PASC encounters at the 2-month and 3-month ahead horizons. Weibull forecasts were more stable than ensemble model forecasts.

**TABLE 3.** Median Model Scores, by Selected Ensemble and Baseline Models

Combination	MAPE	WIS
Ensemble	15.3	322.8
Ensemble (all covariates)	13.9	265.9
Ensemble (all covariates, 3-month lag)	11.3	291.0
Ensemble (all covariates, 6-month lag)	16.2	363.8
Ensemble (PASC encounters)	9.2	206.6
SNaïve	23.2	1,098.3
Weibull 1	11.5	N/A
Weibull 2	9.7	N/A
1 month ahead		
Ensemble	13.6	263.9
Ensemble (all covariates)	8.8	155.6
Ensemble (all covariates, 3-month lag)	6.9	132.9
Ensemble (all covariates, 6-month lag)	15.0	319.6
Ensemble (PASC encounters)	8.8	180.3
SNaïve	28.0	1,082.2
Weibull 1	11.7	N/A
Weibull 2	10.0	N/A
2 months ahead		
Ensemble	18.1	332.9
Ensemble (all covariates)	15.5	378.1
Ensemble (all covariates, 3-month lag)	18.6	438.2
Ensemble (all covariates, 6-month lag)	14.0	326.7
Ensemble (PASC encounters)	12.0	217.3
SNaïve	23.2	1,077.4
Weibull 1	11.5	N/A
Weibull 2	9.7	N/A
3 months ahead		
Ensemble	22.4	410.2
Ensemble (all covariates)	12.7	231.2
Ensemble (all covariates, 3-month lag)	15.1	364.8
Ensemble (all covariates, 6-month lag)	20.4	472.3
Ensemble (PASC encounters)	14.0	277.9
SNaïve	21.4	1,087.8
Weibull 1	11.1	N/A
Weibull 2	9.3	N/A
4 months ahead		
Ensemble	15.2	289.3
Ensemble (all covariates)	4.9	118.4
Ensemble (all covariates, 3-month lag)	12.6	327.5
Ensemble (all covariates, 6-month lag)	45.5	1,488.2
Ensemble (PASC encounters)	5.8	206.6
SNaïve	19.5	1,101.5
Weibull 1	11.5	N/A
Weibull 2	9.7	N/A
5 months ahead		
Ensemble	10.2	227.9
Ensemble (all covariates)	13.7	249.4
Ensemble (all covariates, 3-month lag)	9.2	220.4
Ensemble (all covariates, 6-month lag)	19.2	505.8
Ensemble (PASC encounters)	4.7	200.0
SNaïve	13.7	1,113.7
Weibull 1	12.2	N/A
Weibull 2	10.4	N/A
6 months ahead		
Ensemble	23.9	368.8
Ensemble (all covariates)	30.5	632.3
Ensemble (all covariates, 3-month lag)	11.3	202.2
Ensemble (all covariates, 6-month lag)	32.8	797.0
Ensemble (PASC encounters)	2.9	191.7
SNaïve	18.7	1,326.7
Weibull 1	11.5	N/A
Weibull 2	9.7	N/A

Abbreviations: MAPE, median absolute percent error; WIS, weighted interval score; PASC, post-acute sequelae of COVID-19; SNaïve, seasonal naïve; N/A, not applicable.

**Table 4** shows the results of the full 6-month forecasts. During the forecasting period, from July through December 2022, 23,132 incident cases of PASC were observed. The 6-month lag ensemble model using all covariates had the lowest percent error over the 6-month period at -0.8% (22,960 predicted cases), followed by the unlagged ensemble model using all covariates (+4.3%, 24,174 predicted cases), adjusted Weibull model (-4.7%, 22,093 predicted cases), and the ensemble model using PASC encounters (+5%, 24,353 predicted cases). The seasonal naïve model had the highest percentage error, -71.6%, predicting only 13,479 cases during the 6-month period.

**TABLE 4.** Observed Versus Predicted Six-Month PASC Cases, by Selected Ensemble and Baseline Models

Model	Predicted Cases													
	July 2022		August 2022		September 2022		October 2022		November 2022		December 2022		July-December 2022	
	No.	% Error	No.	% Error	No.	% Error	No.	% Error	No.	% Error	No.	% Error	No.	% Error
Observed	3,702	—	4,712	—	4,163	—	3,938	—	3,553	—	3,064	—	23,132	—
Ensemble	4,368	+15.2	5,567	15.4	5,191	+19.8	4,537	+13.2	3,777	+5.9	3,797	+19.3	27,237	+15.1
Ensemble (all covariates)	3,884	+4.7	3,980	-18.4	4,097	-1.6	4,132	+4.7	4,082	+13.0	4,000	+23.4	24,174	+4.3
Ensemble (all covariates, 3 month lag)	3,510	-5.5	3,352	-40.6	3,710	-12.2	3,444	-14.4	3,557	+0.1	3,409	+10.1	20,982	-10.2
Ensemble (all covariates, 6 month lag)	2,553	-45.0	4,328	-8.9	4,307	+3.3	4,012	+1.8	3,692	+3.8	4,068	+24.7	22,960	-0.8
Ensemble (PASC encounters)	3,359	-10.2	4,874	+3.3	5,509	+24.4	4,168	+5.5	3,466	-2.5	2,976	-2.9	24,353	+5.0
SNaïve	2,491	-48.6	599	-686.3	1,029	-304.6	3,172	-24.2	3,697	+3.9	2,491	-23.0	13,479	-71.6
Weibull 1	4,311	+14.1	4,152	-13.5	3,889	-7.0	3,516	-12.0	3,096	-14.8	2,711	-13.0	21,675	-6.7
Weibull 2	4,387	+15.6	4,229	-11.4	3,964	-5.0	3,586	-9.8	3,159	-12.5	2,768	-10.7	22,093	-4.7

Abbreviations: PASC, post-acute sequelae of COVID-19; No., number; SNaïve, seasonal naïve.

## Discussion

**SUPPLEMENTARY FIGURE 2.**
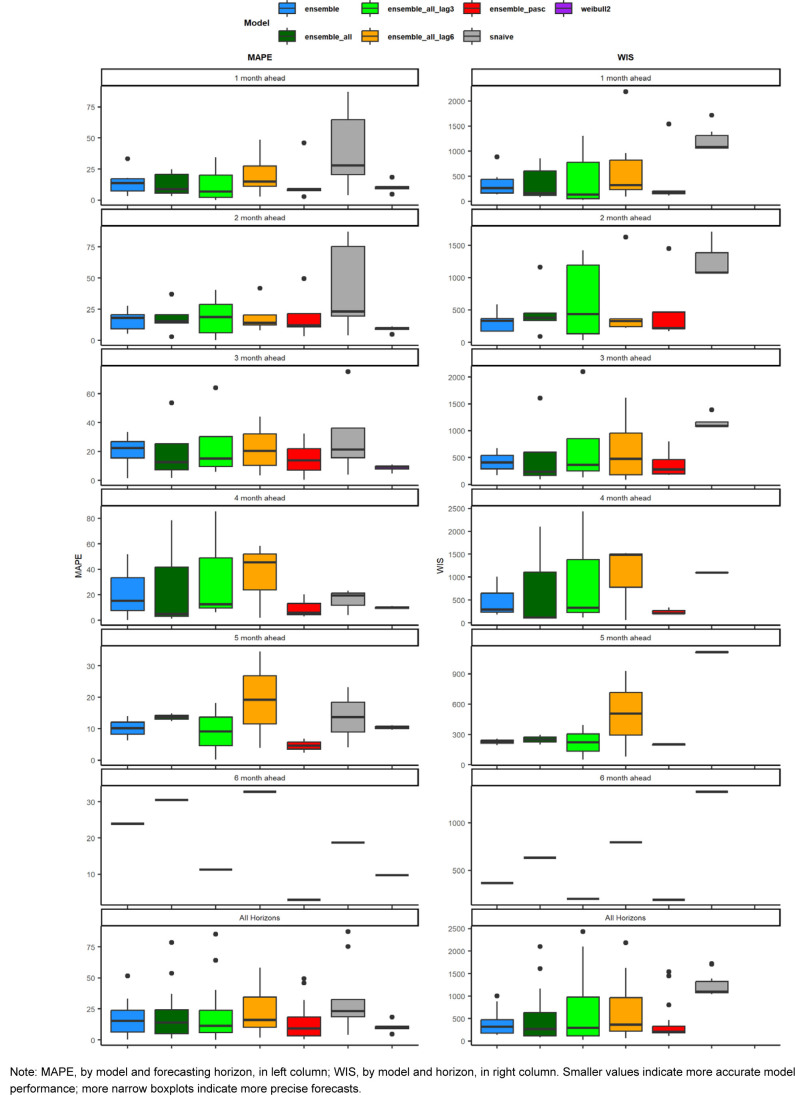
Observed Versus Median Fitted Prediction, by Training Covariates and Data Lag

This study aimed to use various forecasting models, including time series and machine learning models, as well as simple time-based distributions, to predict the number of incident long COVID diagnoses over a 6-month period utilizing various case, outpatient encounter, and demographic data. Forecasts were generated at the beginning of the study period for the entire 6-month period, and 1- to 6-month forecasts were generated for each month in the study period. Monthly forecasts ranged in accuracy, with the PASC encounter ensemble model having the lowest WIS for all forecasting horizons and a MAPE below 10%, as did the adjusted Weibull forecasts, which can be seen in **Table 4** and **Supplementary Figure 2** . No pattern was seen for the 1- to 6-month ahead horizons, with some models performing better at earlier horizons and some performing better at later horizons. This contrasts with COVID-19 forecasts, which tend to perform worse as horizons increase. ^11^

Because long COVID is not an infectious process, it may not be useful to generate monthly forecasts of long COVID diagnoses, but instead generate forecasts for a specified period, to assist senior leaders and public health practitioners with planning for expected case burdens. To this end, forecasts of the entire 6-month period may be most useful. The ensemble model using all covariates and a 6-month lag was the most accurate, with a percent error of just -0.8% (-172 cases) over the study period. This is not unexpected, as the average time to a long COVID diagnosis was 5 months, so lagging covariate data by 6 months is a reasonable choice. Other models also had a percentage error within 5%, however, including the unlagged ensemble model using all covariates (+4.3%), the adjusted Weibull model (-4.7%), and the ensemble model using PASC encounters (+5.0%). These results are similar to estimates in a previous study of Lyme disease, another slow-developing disease. ^8^ Despite having the best model fit using the training data, the 3-month lag all-covariate ensemble model had a percentage error of -10.2%, ranking only sixth best of the 8 models tested. This was not unexpected, as the lag in the full cohort was 5 months, which is closer to the 6-month lag model. It does not explain why the model performed worse than the unlagged ensemble model, however.

This study serves as a ‘proof of concept’ for long COVID forecasting, demonstrating how forecasting models can be used to predict incident long COVID cases up to 6 months in advance, utilizing clinical and demographic data. The study employed existing datasets and surveillance databases to accurately predict the numbers of long COVID diagnoses over a 6-month period.

This study has several limitations. First, models were only trained on COVID-19 cases from January 1, 2020 through June 30, 2022 and, therefore, do not reflect trends in long COVID in later years. Second, the study included the entire U.S., which may not be as useful as regional or single installation forecasts, a possible goal of future studies. Lastly, longer-term horizons, such as the 5- and 6-month forecasts, were limited to just 1 or 2 data points for each model, potentially limiting assessment of those horizons. Future research could focus on the utility of longer-term forecasts by expanding the study period to allow additional forecasts. Additional lag periods, such as the 5-month lag used for the baseline models, can be explored for the ensemble model forecasts.

This study demonstrates that accurate forecasting of long COVID incidence is possible, utilizing clinical, laboratory, and demographic data. Further research needs to determine if results are consistent in more recent time periods, and whether additional or more complex models improve accuracy.
